# Palpebral Tarsal Solitary Neurofibroma

**DOI:** 10.4274/tjo.galenos.2019.47124

**Published:** 2019-09-03

**Authors:** Bülent Yazıcı, Sertaç Argun Kıvanç, Uğur Yayla, Şaduman Balaban Adım

**Affiliations:** 1 Uludag University Faculty of Medicine, Department of Ophthalmology, Bursa, Turkey; 2 Uludag University Faculty of Medicine, Department of Pathology, Bursa, Turkey

**Keywords:** Eyelid, neurofibroma, tarsus, tumor

## Abstract

Solitary neurofibroma is a rare, benign tumor of the peripheral nerve sheath, and is often associated with neurofibromatosis type 1. Herein, a case of palpebral tarsal solitary neurofibroma in a patient without neurofibromatosis is presented, with a review of the literature. A 68-year-old man presented with a subcutaneous mass in the right upper eyelid of 6 months’ duration. Eversion of the eyelid revealed a round, reddish mass on the lateral part of the tarsal plate which measured 12x8 mm in size. The lesion was excised with its tarsal base, diagnosed histologically, and did not recur during a follow-up of 34 months. Isolated, solitary neurofibroma of the eyelid has been reported in a total of 7 cases, including the case presented herein. The tumors arose from the eyelid margin in 4 cases, from the tarsal plate in 2 cases, and from the supratarsal conjunctiva in 1 case. The tumor did not recur after surgical excision in 5 cases for which follow-up data were available.

## Introduction

Neurofibromas are benign peripheral nerve sheath tumors and are usually associated with neurofibromatosis type 1 (NF1). Morphologically, there are plexiform, solitary (local or isolated), and diffuse subtypes. The most common subtype in the periocular area is NF1-associated plexiform neurofibroma.^1^ Solitary neurofibroma (SN) not associated with NF1 is rare in the eyelid and conjunctiva. There is only one previously reported case of SN in the eyelid tarsal plates.^2^

## Case Report

**Figure 1:**
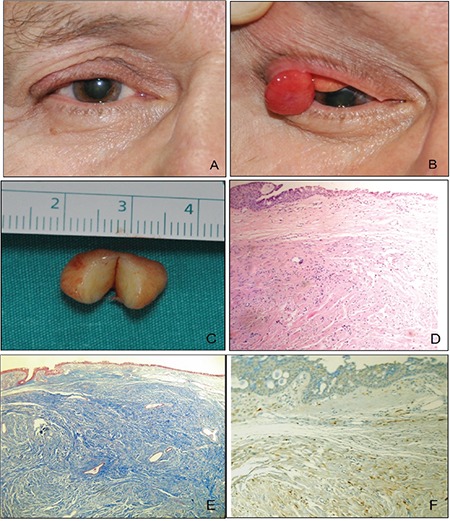
A) The patient presented with a painless subcutaneous mass located on the lateral side of the right upper eyelid; B) A solitary neurofibroma originates from the tarsal plate of the upper eyelid; C) Appearance of the tumor after bisection; D) Tumor tissue consisted of spindle-shaped, well-demarcated cells in the conjunctiva, with comma- or fusiform-shaped nuclei (Hematoxylin-eosin, x100); E) Masson’s trichrome stain revealed intense collagenization in the tumor stroma (blue, x100); F) Immunohistochemical staining in tumor cells showing S100 positivity and indicating neural origin (x100)

A 68-year-old man presented with a 6-month history of painless subcutaneous mass in his right upper eyelid (Figure 1A). Eversion of the eyelid revealed a round, reddish mass attached to the lateral tarsus by a short peduncle (Figure 1B). The ocular examination was otherwise unremarkable. The patient had no symptoms or history of NF1.

The lesion was excised together with its tarsal base under local anesthesia. The tarsal defect was left to heal by secondary intention. The tumor was 12x8 mm in size and hard in consistency (Figure 1C). Histologically, the tumor consisted of spindle-shaped peripheral nerve sheath cells and a collagenous stroma (Figure 1D). Masson’s trichrome staining showed dense collagen fibers around the neoplastic cells (Figure 1E). Immunohistochemically, the tumor cells were positive for S100 and negative for smooth muscle actin protein and desmin (Figure 1F). These findings were consistent with SN. No postoperative complications were observed; there was no recurrence of the tumor during the 34-month follow-up period.

## Discussion

Solitary, benign peripheral nerve tumors not associated with NF1 can be classified as traumatic neuroma, SN, and schwannoma. Solitary neurofibromas occur most frequently in adults, preferentially affecting males and presenting as subcutaneous masses on the extremities and trunk.^3^ In the periocular region, SNs mostly originate from intraorbital nerves and particularly in the superior-posterior orbit.^4^

We found 6 cases of isolated eyelid SN in the literature.^2,5,6,7,8,9^ Including the present case, 2 of the total 7 patients were male. One patient was 14 years old,^7^ and the ages of the other patients varied between 39 and 81 years. The tumor was located in the upper eyelid in 5 patients: in the tarsus in 2 patients (including the present case), the eyelid margin in 2 patients, and at supratarsal conjunctiva in 1 patient.^2,6,8,9^ In 2 patients, the lesions were in the lower eyelid and near the lacrimal punctum and lateral canthus.^5,7^ The time from noticing the lesion to surgical excision ranged between 6 months and 5 years, and was not specified in one case.^6^ In 3 cases, the lesion was mistaken for chalazion.

Our case was macroscopically similar to the tarsal SN described by Shibata et al.^2^ In both cases, the lesion was round, hard, and located at the lateral aspect of the upper tarsus. The center of the lesion was more vascularized and slightly depressed. As in the earlier cases of eyelid SN, we were unable to identify the specific nerve that gave rise to our patient’s tumor.

Concurrent systemic diseases in different patients included lymphoma^2^, lung adenocarcinoma,^6^ and Sjögren’s syndrome.^7^ In one patient, the tumor was associated with basal cell carcinoma of the eyelid.^6^ Including the case presented here, tumor recurrence was not observed in a total of 5 patients during follow-up of 2-36 months after surgical excision.^2,5,8^ There were no follow-up data for 2 patients.^6,9^

Preoperative diagnosis of such a rare condition is challenging. However, the macroscopic features of the tarsal SNs in 2 patients were quite different from those of common tarsal masses such as chalazion and meibomian gland carcinoma. Schwannoma, leiomyoma, and malignant peripheral nerve sheath tumors must be included in the histological differential diagnosis of SN. Tumors of muscular origin are positive for desmin and smooth muscle actin proteins, while tumors of neural origin are positive for S100. Like neurofibromas, schwannomas are also positive for S100. However, they stain more intensely because neurofibromas have a more complex structure that includes Schwann cells, perineural cells, and fibroblasts. More cases are needed to better characterize tarsal SNs.
